# The Detection of Metabolite-Mediated Gene Module Co-Expression Using Multivariate Linear Models

**DOI:** 10.1371/journal.pone.0150257

**Published:** 2016-02-26

**Authors:** Trishanta Padayachee, Tatsiana Khamiakova, Ziv Shkedy, Markus Perola, Perttu Salo, Tomasz Burzykowski

**Affiliations:** 1 Interuniversity Institute for Biostatistics and Statistical Bioinformatics (I-Biostat), Hasselt University, Diepenbeek, Belgium; 2 Unit of Public Health Genomics, National Institute for Health and Welfare, Helsinki, Finland; National Institute of Genomic Medicine, MEXICO

## Abstract

Investigating whether metabolites regulate the co-expression of a predefined gene module is one of the relevant questions posed in the integrative analysis of metabolomic and transcriptomic data. This article concerns the integrative analysis of the two high-dimensional datasets by means of multivariate models and statistical tests for the dependence between metabolites and the co-expression of a gene module. The general linear model (GLM) for correlated data that we propose models the dependence between adjusted gene expression values through a block-diagonal variance-covariance structure formed by metabolic-subset specific general variance-covariance blocks. Performance of statistical tests for the inference of conditional co-expression are evaluated through a simulation study. The proposed methodology is applied to the gene expression data of the previously characterized lipid-leukocyte module. Our results show that the GLM approach improves on a previous approach by being less prone to the detection of spurious conditional co-expression.

## 1 Introduction

Omics technologies have rapidly advanced giving rise to an extensive amount of omics (genomics, proteomics, metabolomics, transcriptomics, glycomics, and lipidomics) data with widespread availability. To obtain a comprehensive understanding of complex diseases, research is now centring on the integrative analysis of omics data, necessitating more advanced methodological frameworks. In this article, we focus on the integrative analysis of metabolomic and transcriptomic data to investigate the co-expression of a gene module (a set of co-expressed (correlated) genes belonging to the same biological pathway) conditional on metabolic concentrations.

Conditional co-expression is the observation of dependence of the correlation(s) (or other measure(s) of association) of gene expression levels on values of a covariate. It is investigated to gain insight into the regulatory mechanisms resulting in gene co-expression and, in turn, to gain insight into the mechanisms of complex diseases. In this article, we use the term conditional co-expression, though the term differential co-expression is also often used to describe the phenomenon of regulated co-expression [1]. Based on their intent, investigations of conditional co-expression fall under two distinct categories. A targeted study focuses on a predefined set of highly co-expressed genes, termed a gene module, and investigates whether it is conditionally co-expressed. An untargeted/semi-targeted study considers all genes and attempts to identify conditionally co-expressed gene modules [2]. Several studies investigated the co-expression of gene pairs or gene modules between two biological conditions such as diseased and healthy, young and old, male and female, or between two species such as humans and chimpanzees [2]. For instance, a gene pair that is strongly correlated in healthy samples and weakly correlated in diseased samples (or *vice-versa*) exhibits a pattern of conditional co-expression. Similarly, pairs of genes from a conditionally co-expressed gene module have correlation coefficients (or other measures of association) which differ across certain biological conditions.

A wide range of methods have been proposed for the detection of conditionally co-expressed gene pairs and gene sets, particularly across two biological conditions. Kayano et al. (2014) [1] review the methods for the detection of conditionally co-expressed gene pairs characterized by cross, i.e., a biological phenomenon in which two genes are positively correlated under one condition and negatively correlated under the other condition. Methods to detect gene sets with positive correlations under one condition and random gene-pair correlations under the other condition are also reviewed. In the review, the need for more efficient techniques is highlighted. Differential co-expression network analysis is one of the more commonly implemented techniques for the detection of conditional co-expression [3, 4]. Fewer methodologies have been proposed for the investigation of co-expression across multiple groups. Gillis and Pavlidis (2009) [5] analyzed co-expression across multiple-ordered groups (defined by age categories). Chen et al. (2011) [6] proposed a penalized-likelihood approach for bivariate conditional normal models to identify variables that mediate the co-expression of a gene pair.

We focus on a targeted conditional co-expression analysis, i.e., the investigation of an a priori defined gene module with the aim of identifying variables that mediate its co-expression. Our study is motivated by the conditional co-expression analysis presented in [7]. Inouye et al. (2010) [7] provide a proof-of-concept paper for the integrative analysis of metabolomic, transcriptomic, and genomic data. In particular, they explore the serum-metabolite mediation of the recently characterized core Lipid-Leukocyte (LL) gene module’s [8] co-expression. Toward this aim, they fit a simple linear regression model to Spearman’s correlation coefficients for all pairs of genes of the core LL module for five subsets of samples formed by using quintiles of the metabolite concentrations. In this way, the dependence of the correlation (co-expression) on metabolic concentrations can be detected and quantified.

The method applied by [7], although innovative, is limited in several aspects:

1. It does not allow for the adjustment of the gene expression values for potential confounding factors. As a consequence, relevant correlations can be missed or spurious correlations can be detected.
2. The simple linear model framework incorrectly treats the correlation coefficients as independent. In addition, the estimation error in the coefficients is ignored.
3. The approach focuses only on linear trends in co-expression by metabolic concentrations.
4. The results may depend on the definition of metabolic subsets.

In this paper, we consider a modeling approach that addresses points 1–3 from the aforementioned list. In particular, we use a general linear model (GLM) for correlated data [9, 10] to analyze the dependence structure of gene expression measurements for different metabolic subsets. Statistical tests for the inference of conditional co-expression are proposed. A simulation study is conducted to evaluate the Type I error probability and the sensitivity of the test statistics to different co-expression dynamics. We apply the model to a subset of the DILGOM (Dietary, Lifestyle, and Genetic determinants of Obesity and Metabolic syndrome) study data collected in Helsinki, Finland to study the serum metabolite-induced conditional co-expression for the core LL module.

The paper is organized as follows. Section 2 introduces the data. Section 3 describes the statistical methodology and the workflow of the analysis. Results of the simulation study and the DILGOM analysis appear in Section 4. The discussion and conclusions are presented in Section 5.

## 2 Data

We analyze the complete cases of a subset of participants from the Helsinki population-based cohort recruited in the DILGOM study [8]. The individuals in the subset were assessed for metabolomic, genome-wide transcriptomic, and genomic variation. Serum metabolite concentrations were measured using proton NMR spectroscopy. Gene expression data were obtained from blood lymphocytes using the Illumina HT-12 expression array (Illumina Inc., San Diego, CA, USA). We use the phenotypic data on age and gender, the metabolomic data, and the transcriptomic data of the core LL gene module. Of the complete case observations (*N* = 466), 215 correspond to males and 251 to females, with age ranging from 25 to 74 years.

### 2.1 Metabolomic data

**Fig 1:**
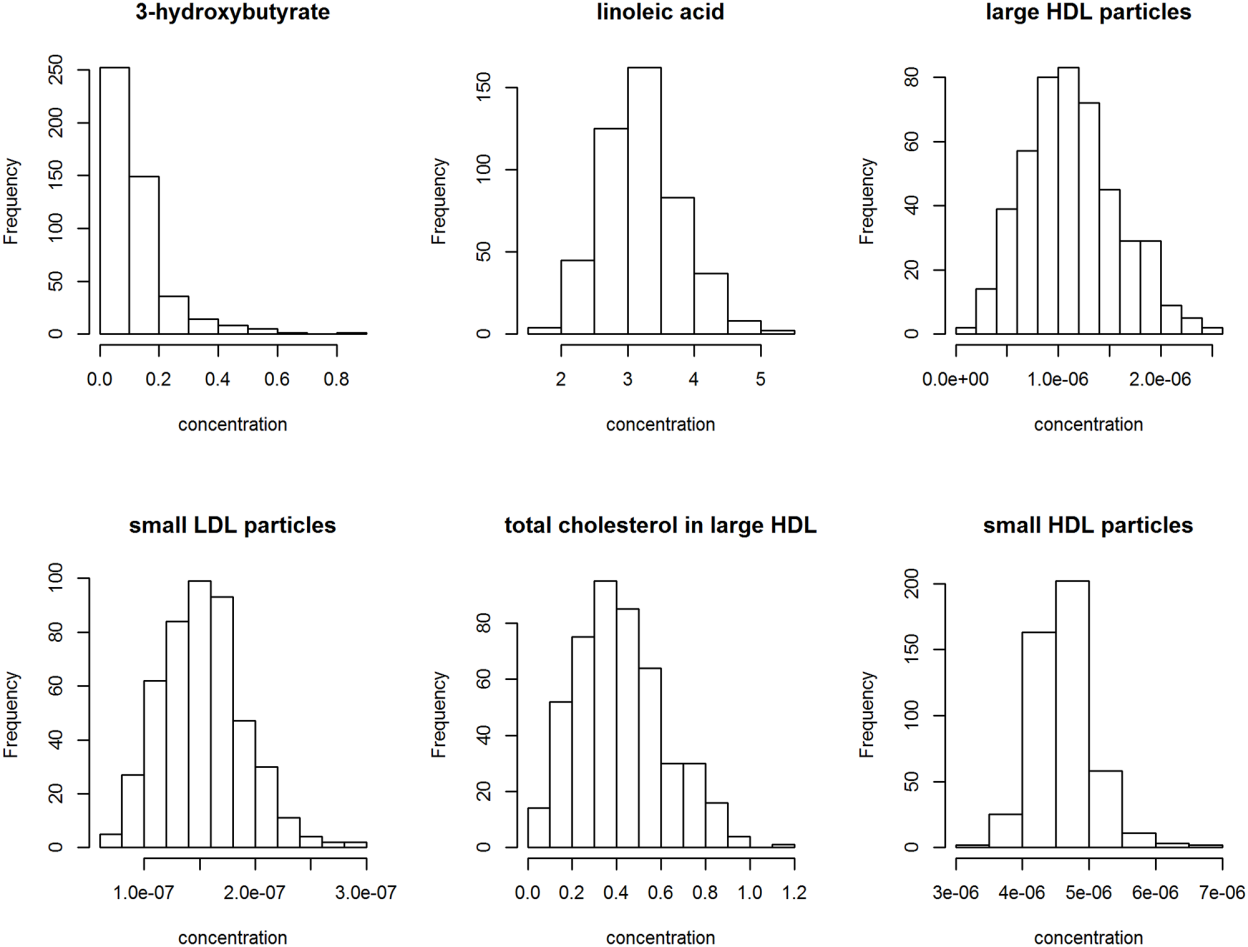
Histograms of the observed values for 3-hydroxybutyrate, linoleic acid, large HDL particles, small HDL particles, small LDL particles, and total cholesterol in large HDL.

Metabolomic data were available on 137 serum metabolites inclusive of amino acids, lipids, and sugars. For illustration we primarily focus on six metabolites: 3-hydroxybutyrate, linoleic acid, large HDL particles, small HDL particles, small LDL particles, and total cholesterol in large HDL as in [7]. Histograms of the observed values of these metabolites are shown in Fig 1, with summary statistics listed in Table 1. Due to the non-normality of the distributions, metabolic concentrations were transformed using the two-parameter Box-Cox transformation [11]. The normalized metabolite distributions were then corrected for age, gender, and their two-way interaction using metabolite-specific ANOVA models.

**Table 1:** Summary statistics of the observed concentrations for the six metabolites selected for illustration (N = 466).

metabolite	mean	standard deviation	1^st^ quartile	median	3^rd^ quartile
3-hydroxybutyrate	0.1290	0.0970	0.0768	0.0955	0.1363
linoleic acid	3.2141	0.5879	2.8233	3.1735	3.5635
large HDL particles (×10^−6^)	1.1334	0.4531	0.8076	1.1080	1.4133
small LDL particles (×10^−6^)	0.1524	0.0373	0.1249	0.1504	0.1745
total cholesterol in large HDL	0.4157	0.2008	0.2696	0.3961	0.5418
small HDL particles (×10^−6^)	4.6213	0.4505	4.3520	4.6075	4.8668

### 2.2 Transcriptomic data

The LL gene module is comprised of 11 highly correlated genes. Seven of these genes—HDC, FCER1A, GATA2, CPA3, MS4A2, SPRYD5 and SLC45A3—form the core LL gene module [8]. The LL module is of interest as it harbours key immune response mediators and is strongly associated with serum lipid concentrations [7] linking it to the two main contributors of coronary artery disease (CAD), namely, inflammation [12] and lipids (such as high density lipoprotein (HDL) and low density lipoprotein (LDL)).

**Fig 2:**
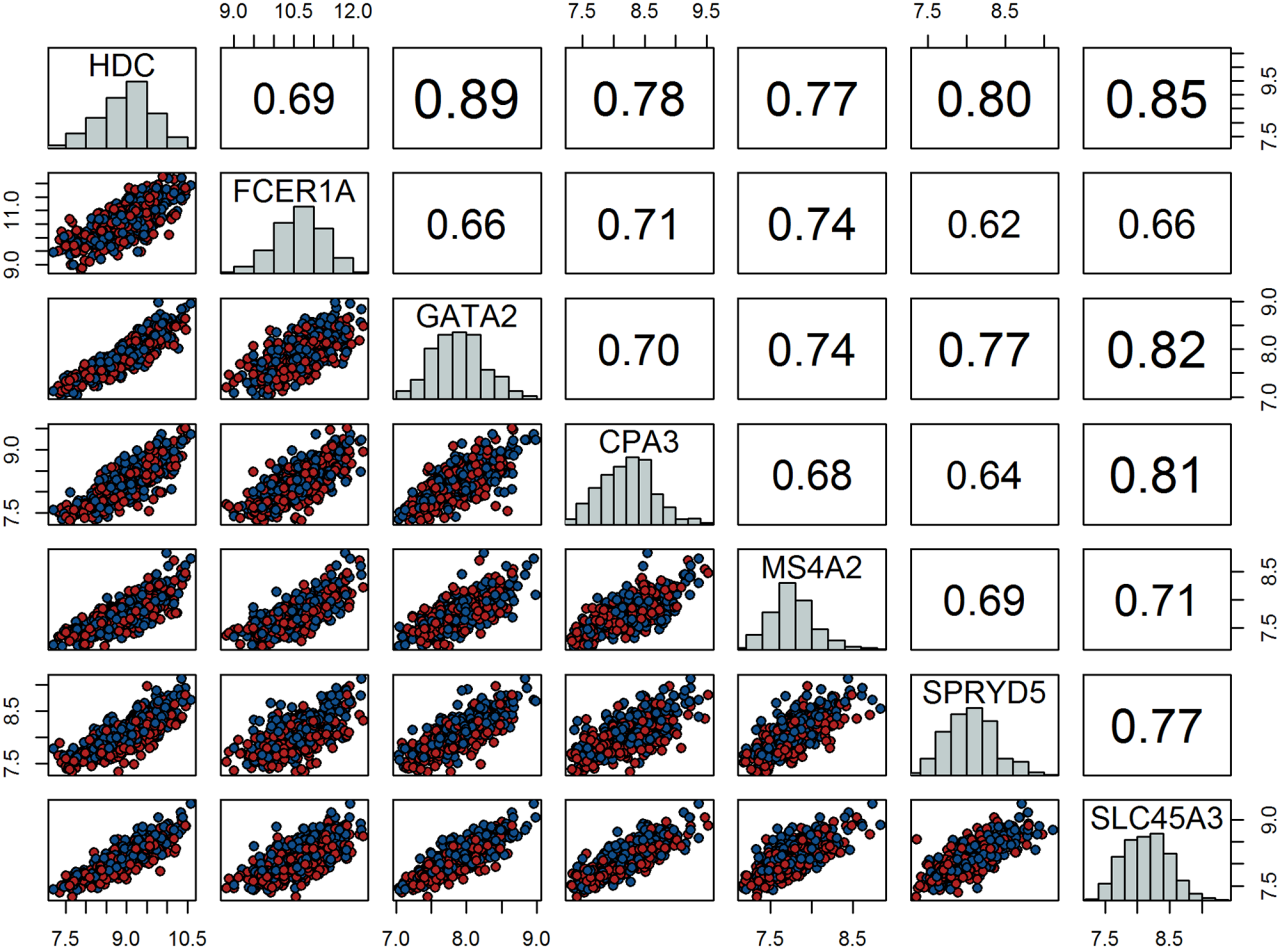
Scatter-plot matrix of the core LL module gene expression values. Scatter-plots of the expression values for each gene pair appear in the lower triangular matrix. Points are colour coded by gender: red represents males and blue represents females. Pairwise Spearman’s correlation coefficients are indicated in the upper triangular matrix. The distribution of gene expression values for each gene is illustrated on the main diagonal.

**Fig 3:**
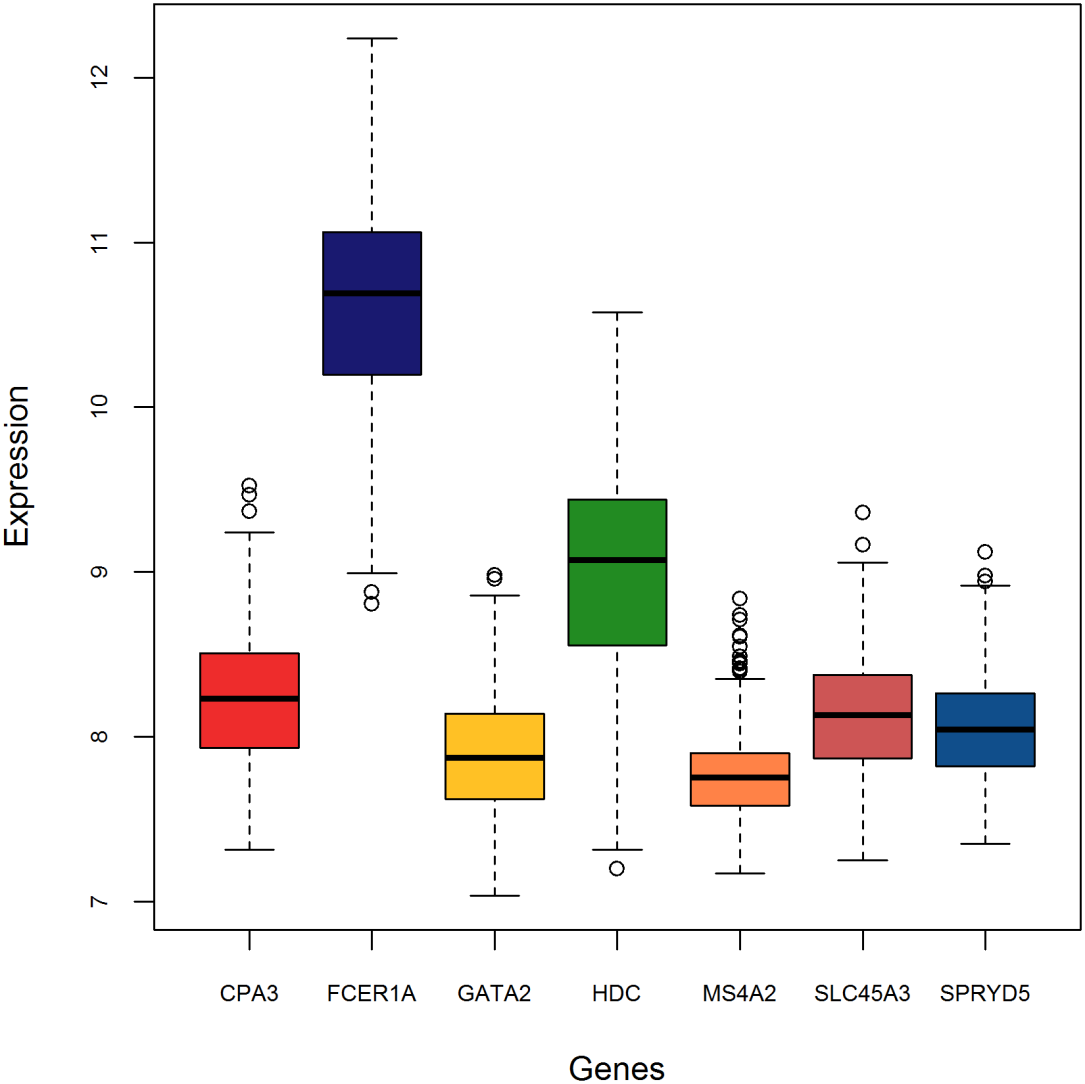
Box-plots of the core LL module expression. Heterogeneous mean expression values and variances are observed.

Genes forming the core LL module are highly correlated (see Fig 2), with Spearman’s correlation coefficients larger than 0.6, and they have heterogeneous variances (see Fig 3).

The gene expression data were normalized using quantile normalization and quality control was performed as described in [8].

## 3 Statistical Methodology

### 3.1 Exploratory analysis

To get a general idea of the co-expression dynamics as a function of metabolic concentrations, we estimate sliding-window correlations. In preparation, for a specific metabolite, the data are sorted in ascending order of the observed metabolic concentrations and a window size (expressed as a proportion, represented by *w*, of the total sample size) is selected. The procedure begins by computing Spearman’s correlation coefficients between pairs of genes for the first *w* × *N* individuals, together with the corresponding mean metabolite value. Then, the window is shifted so that it starts from the second ordered metabolite measurement, and the window-specific correlation coefficients and mean metabolite value are estimated. The procedure continues until the window includes the last (ordered) metabolite measurement. The obtained correlation coefficients are plotted against the mean metabolite values. The smoothness of the plot depends on the window size: selecting a large window results in a smoother estimate of the correlation trajectory.

### 3.2 Simple linear regression of Spearman’s correlation coefficients

The conditional co-expression analysis by [7] is performed per metabolite. For a given metabolite, the data are split into five subsets based on quintiles of the metabolite’s concentration. For each subset, Spearman’s rank correlation coefficients are computed for all pairs of genes in the core LL module. A linear regression model is used to relate the estimated correlation coefficients to the quintiles upon which the metabolic subsets are defined.

Using a formal notation, the following model is fitted:

$$\begin{array}{c} Y_{sp}=\alpha +\beta x_{s}+\varepsilon_{sp}, \end{array}$$ (1)

where *s* (*s* = 1, …, *S*) indexes the metabolic subsets (*S* = 5 for our case study), *p* (*p* = 1, …, *G*(*G* − 1)/2) indexes the gene pairs with *G* denoting the number of genes in the gene module (*G* = 7 for the core LL gene module), *Y*_*sp*_ is the Spearman’s correlation coefficient for the *p*-th gene-pair in the *s*-th metabolic subset, and *x*_*s*_ is the value of the *s*-th quintile of the metabolic concentration. As in classical linear regression, *ε*_*sp*_ are residual errors that are assumed to be independent and normally distributed with mean zero and variance $\sigma_{e}^{2}$.

To determine whether there is a relationship between the module co-expression and the metabolite concentrations, the null hypothesis of a zero slope, *H*_0_ : *β* = 0, is tested against the alternative hypothesis, *H*_*A*_ : *β* ≠ 0.

### 3.3 General linear model (GLM) for gene expression measurements

In accordance with the simple linear-regression approach, this analysis is performed per metabolite. For a given metabolite, the data are split into five metabolic-subsets based on quintiles of the metabolite’s concentration. Gene expression values are modeled using a GLM allowing for a correlation between an individual’s gene expression values. A general variance-covariance structure of within-individual gene expression measurements is assumed for each metabolic subset.

In a formal notation, the following model is considered:

$$\begin{array}{c} y_{si}=X_{si}\beta +\varepsilon_{si}, \end{array}$$ (2)

where $y_{si}={\left(y_{si1},\ldots ,y_{siG}\right)}^{T}$ is the vector of gene expression measurements for the *i*-th individual (*i* = 1, …, *n*_*s*_) in the *s*-th subset, $X_{si}$ is a *G* × *R*-dimensional matrix of *R* covariates (an example of the design matrix $X_{si}$ is included in the S1 File), *β* is an *R*-dimensional vector of coefficients corresponding to the *R* covariates, and $\varepsilon_{si}$ is a *G*-dimensional vector of residual errors which are normally distributed with zero mean and variance-covariance matrix $\Sigma_{s}$. In particular,

$$\begin{array}{c} \Sigma_{s}=\left(\begin{array}{cccc} \sigma_{s,1}^{2} & \rho_{s,12}\sigma_{s,1}\sigma_{s,2} & \cdots & \rho_{s,1G}\sigma_{s,1}\sigma_{s,G}\\ \rho_{s,12}\sigma_{s,1}\sigma_{s,2} & \sigma_{s,2}^{2} & \cdots & \rho_{s,2G}\sigma_{s,2}\sigma_{s,G}\\ \vdots & \vdots & \ddots & \vdots \\ \rho_{s,1G}\sigma_{s,1}\sigma_{s,G} & \rho_{s,2G}\sigma_{s,2}\sigma_{s,G} & \cdots & \sigma_{s,G}^{2} \end{array}\right), \end{array}$$ (3)

where $\sigma_{s,g}^{2}$ is the variance of the *g*-th gene for the *s*-th subset and *ρ*_*s*, *g*_1_*g*_2__ is the correlation between genes *g*_1_ and *g*_2_ for the *s*-th subset.

The null hypothesis of no metabolite-dependent co-expression can be seen as corresponding to the following variance-covariance structure:

$$\begin{array}{c} \Sigma_{s}^{\left(0\right)}=\left(\begin{array}{cccc} \sigma_{s,1}^{2} & \rho_{12}\sigma_{s,1}\sigma_{s,2} & \cdots & \rho_{1G}\sigma_{s,1}\sigma_{s,G}\\ \rho_{12}\sigma_{s,1}\sigma_{s,2} & \sigma_{s,2}^{2} & \cdots & \rho_{2G}\sigma_{s,2}\sigma_{s,G}\\ \vdots & \vdots & \ddots & \vdots \\ \rho_{1G}\sigma_{s,1}\sigma_{s,G} & \rho_{2G}\sigma_{s,2}\sigma_{s,G} & \cdots & \sigma_{s,G}^{2} \end{array}\right), \end{array}$$ (4)

in which the correlation coefficients *ρ*_*g*_1_*g*_2__ do not depend on the metabolic-subset. In correspondence with $\Sigma_{s}$, the gene variances $\sigma_{s,g}^{2}$ are metabolic-subset specific.

The null hypothesis of no metabolite-dependent co-expression can be tested by using the likelihood-ratio (LR) test comparing the null model specified by Eqs (2) and (4) with the alternative model defined by Eqs (2) and (3). Wilks (1938) [13] showed that the asymptotic distribution of the LR test is a $\chi_{\left(k\right)}^{2}$ distribution where *k* is the difference in the number of parameters estimated between the alternative model and the null model. However, there is evidence suggesting that the approximation to a chi-squared distribution may be rather poor for small sample sizes [14][15].

The statistical test proposed by Larntz & Perlman (1985) [16] is a possible alternative to the LR test for testing the equality of correlation matrices. In the Larntz & Perlman approach, each of the *G*(*G* − 1)/2 hypotheses of equal correlations (i.e., *H*_*g*_1_*g*_2__ : *ρ*1, _*g*_1_*g*_2__ = *ρ*2, _*g*_1_*g*_2__ = … = *ρ*_*S*, *g*_1_*g*_2__ for all *g*_1_ ≠ *g*_2_ (*g*_1_, *g*_2_ = 1, …, *G*)) is tested by using the statistic

$$\begin{array}{c} S_{g_{1}g_{2}}=\sum_{i=1}^{S}\left(n_{s}-3\right)z_{s,g_{1}g_{2}}^{2}-\frac{{\left[\sum_{i=1}^{S}\left(n_{s}-3\right)z_{s,g_{1}g_{2}}\right]}^{2}}{\sum_{i=1}^{S}\left(n_{s}-3\right)} \end{array}$$ (5)

where *z*_*s*, *g*_1_*g*_2__ is the Fisher’s z-transformed correlation between genes *g*_1_ and *g*_2_ for the *s*-th subset. To test the equality of the correlation matrices, the composite test statistic *T*, defined as the maximum of the *G*(*G* − 1)/2 test statistics, is computed:

$$\begin{array}{c} T=\text{max}\;S_{g_{1}g_{2}}\;\text{for}\;1\leq g_{1}<g_{2}\leq G \end{array}$$ (6)

Under the null hypothesis, *T* has an asymptotic *χ*^2^ distribution with *S* − 1 degrees of freedom. The Sidák inequality is used to control the probability of committing a Type I error. As such, the null hypothesis of no metabolite-dependent co-expression is rejected if

$$\begin{array}{c} T>\chi_{S-1,\alpha^{{}^{\prime }}}^{2} \end{array}$$ (7)

where *α*′ = 1 − (1 − *α*)^2/*G*(*G* − 1)^ is the Sidák-adjusted significance level. The Larntz & Perlman approach has been reported to have good small-sample properties as it relies on the univariate normality of the Fisher’s z-transformed correlations [16].

Other possible statistical approaches for testing the equality of correlation matrices include the statistical tests proposed by Cole (1968) [17] and Jennrich (1970) [18] which are based on a quadratic form of deviations from the mean and have an asymptotic *χ*^2^ distribution with (*S* − 1)*G*(*G* − 1)/2 degrees of freedom [19].

### 3.4 Multiple comparisons *p*-value adjustment

The simple linear regression approach (Section 3.2) and the GLM approach (Section 3.3) both entail fitting a separate model per metabolite. Hence, a multiple testing adjustment should be considered to control either the family-wise error rate (FWER) or the false discovery rate (FDR). FWER-controlling procedures restrict the probability of committing a Type I error (i.e., falsely rejecting the null hypothesis for any of the tests conducted). Controlling the FDR is a less stringent, and hence more powerful, approach that instead controls the proportion of discoveries that are allowed to be false. Given the correlated nature of our hypothesis tests (i.e., due to the correlation within the metabolomics data), we chose the Benjamini and Yekutieli FDR-controlling procedure [20]. It is an extension of Benjamini and Hochberg’s correction for cases where the independence of hypothesis tests cannot be assumed [20]. Lin et al. (chapter 6) [21] discuss an assortment of FDR-controlling procedures and their implementation using the R statistical programming language.

### 3.5 Workflow

#### Simulation study

**Fig 4:**
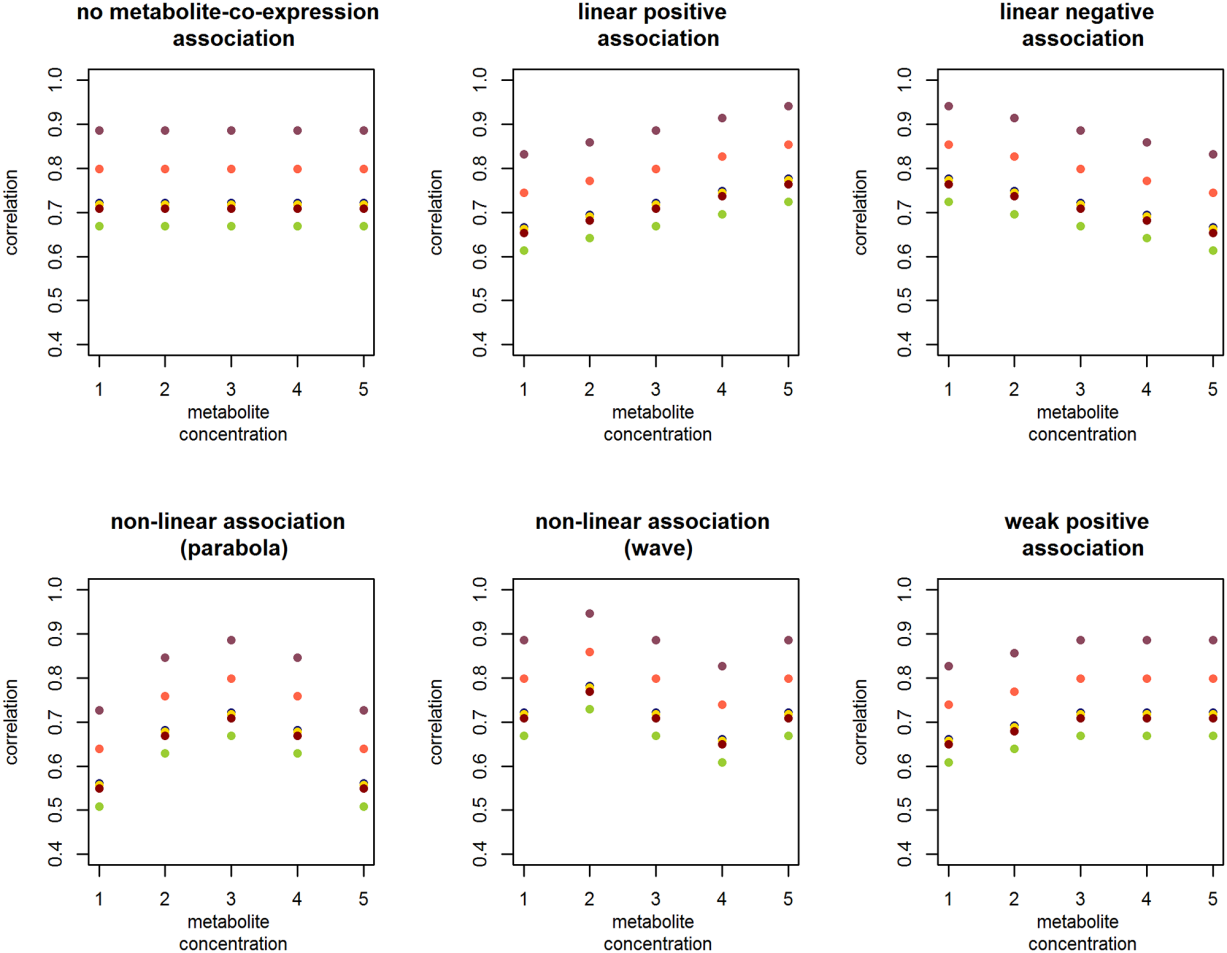
The six simulated co-expression dynamics for a four gene module. The four genes of the simulated module generate six gene-pair correlations. Each trajectory of dots captures the metabolite-co-expression association for one of the module gene pairs.

To assess the Type I error probability and the power of the proposed GLM methodology for different co-expression dynamics, we simulate data reflecting six variations in metabolite-co-expression dependence (Fig 4). Specifically, we simulate:

- data characterised by no metabolite-co-expression dependence,
- data based on an approximately linear positive association between co-expression and metabolic concentrations and another dataset based on an approximately linear negative metabolite-co-expression association,
- data based on two variations of non-linear dependencies, and
- data exhibiting a weak positive metabolite-co-expression association.

For each of the six co-expression dynamics, we create 1000 datasets of 125, 450, and 800 observations each. Metabolic concentrations are sampled from a normal distribution with mean 3.2141 and variance 0.3456 (i.e., the distribution of linoleic acid in the DILGOM subset). Gene expression values are sampled from a multivariate normal distribution with means and variances corresponding to that of the CPA3, FCER1A, GATA2, HDC, MS4A2, SLC45A3, and SPRYD5 expression values in the DILGOM data. Gene-pair correlations vary with the metabolite concentration in a manner defined by one of the six metabolite-co-expression associations listed above. These co-expression dynamics are illustrated in Fig 4. To investigate the Type I error probability, data (i.e., characterised by no metabolite co-expression dependence) are simulated for a four, five, and seven gene module. Data for the power investigation are simulated for a module of four genes. The linear regression model and the GLM-based LR, Larntz & Perlman, Jennrich, and Cole tests are applied to the simulated data (see Section 3.3).

#### DILGOM analysis

Using the DILGOM data, described in Section 2, we study the metabolite co-expression association by means of the GLM for gene expression values (Section 3.3) and the linear-regression approach of Inouye et al. [7] (Section 3.2). The mean structure of the GLM, defined in Eq (2), included the four-way interaction between gene, (the Box-Cox transformed and age-gender interaction adjusted) metabolite concentration, age, and gender. The *p*-values of the metabolite-specific tests were adjusted by using the Benjamini and Yekutieli [20] FDR-controlling procedure.

#### Practical implementation

The GLMs were fitted using PROC GLIMMIX of SAS 9.4. The COVTEST statement of PROC GLIMMIX enables the statistical inference on covariance parameters. The LR test is implemented by specifying constraints in the COVTEST statement that, when applied to the variance-covariance structure of the alternate model Eq (3), defines the null model’s variance-covariance structure Eq (4). The generic SAS code is provided as supporting information (S1 SAS Code). For ease of illustration, the included code is for a module of three genes. Functions to implement the Larntz & Perlman (1985), Jennrich (1970), and Cole (1968) tests were coded in the R programming language. The Benjamini and Yekutieli adjustment was performed using R 3.1.1 and the R-package *multtest*.

## 4 Results

### 4.1 Simulation study

We have found that the Larntz & Perlman test statistic outperforms the Jennrich and Cole statistics with regard to the proper control of the Type I error probability. Thus, in what follows, we will focus on the linear-regression approach, the GLM-based LR test and the GLM-based Larntz & Perlman test. The results of the GLM-based Jennrich and Cole statistics are shown in S1 and S2 Tables.

Table 2 integrates the simulation results for the investigation of the Type I error probability. The linear-regression approach fails to control the Type I error probability. When the sample size is small (*n* = 125), the Type I error probability becomes unacceptably high. On the other hand, for large sample sizes (relative to the number of estimated correlation coefficients), the linear regression becomes too conservative. Due to these extreme fluctuations in the Type I error probability, the linear regression approach cannot be deemed a reliable analysis method, as it is difficult to know in a practical setting whether the regression-based test will be liberal or conservative. The GLM-based LR test provides better control of the Type I error probability than the linear-regression approach, particularly for large sample sizes (i.e., when the asymptotic properties of the LR test come into effect). However, the probability is inflated for small sample sizes. The Larntz & Perlman approach properly controls the Type I error probability, with a slight tendency to become conservative for large sample sizes. Hence, combining the Larntz & Perlman test with a suitable multiple-testing procedure should result in a testing framework that properly controls the FWER or the FDR.

**Table 2:** Type I error probabilities for the linear regression and the GLM-based test statistics by module size and sample size.

module size	sample size (*n*)	linear regression ^⋆^	GLM-based LR test ^⋆^	GLM-based Larntz & Perlman ^⋆^
4	125	0.205 [0.179, 0.231]	0.109 [0.089, 0.129]	0.045 [0.032, 0.058]
4	450	0.056 [0.041, 0.071]	0.062 [0.047, 0.077]	0.043 [0.030, 0.056]
4	800	0.020 [0.011, 0.029]	0.053 [0.039, 0.067]	0.035 [0.023, 0.047]
5	125	0.197 [0.172, 0.222]	0.141 [0.119, 0.163]	0.048 [0.034, 0.062]
5	450	0.064 [0.048, 0.080]	0.067 [0.051, 0.083]	0.037 [0.025, 0.049]
5	800	0.022 [0.012, 0.032]	0.066 [0.050, 0.082]	0.048 [0.034, 0.062]
7	125	0.461 [0.430, 0.492]	0.314 [0.285, 0.343]	0.035 [0.023, 0.047]
7	450	0.314 [0.285, 0.343]	0.083* [0.065, 0.100]	0.036* [0.024, 0.048]
7	800	0.212 [0.186, 0.238]	0.070** [0.054, 0.087]	0.029** [0.018, 0.040]

^⋆^ estimate [95% confidence interval]

* convergence rate of GLM: 0.991

** convergence rate of GLM: 0.993

Table 3 shows the results of the power investigation. In view of the problems with the control of the Type I error probability for the linear-regression test and the GLM-based LR test, we focus on the sensitivity of the test statistics to detect the co-expression dynamics in the case of a four-gene module and a sample size of *n* = 450 observations. This is because for this case the Type I error probability, shown in Table 2, did not differ significantly from 0.05 for the three approaches. Table 3 indicates that the power of the GLM-based LR test and the Larntz & Perlman test is comparable. The GLM-based tests are clearly more powerful than the linear-regression-based test in detecting linear trends and are substantially more powerful in the case of non-linear trends. The only case when the linear-regression-based approach shows some advantage is a weak positive association.

**Table 3:** Power of the linear regression and GLM-based test statistics for different co-expression dynamics and sample sizes.

co-expression dynamics	sample size (*n*)	linear regression ^⋆^	GLM-based LR test ^⋆^	GLM-based Larntz & Perlman ^⋆^
linear positive association	125	0.408 [0.377, 0.439]	0.314 [0.285, 0.343]	0.188 [0.163, 0.213]
linear positive association	450	**0.635** [0.605, 0.665]	**0.826** [0.802, 0.850]	**0.797** [0.772, 0.822]
linear positive association	800	0.712 [0.683, 0.741]	0.990 [0.983, 0.997]	0.989 [0.982, 0.996]
linear negative association	125	0.451 [0.420, 0.482]	0.300 [0.271, 0.329]	0.184 [0.159, 0.209]
linear negative association	450	**0.621** [0.590, 0.652]	**0.838** [0.815, 0.861]	**0.819** [0.795, 0.843]
linear negative association	800	0.723 [0.695, 0.751]	0.988 [0.981, 0.995]	0.987 [0.979, 0.995]
non-linear association (parabola)	125	0.219 [0.193, 0.245]	0.293 [0.264, 0.322]	0.243 [0.216, 0.270]
non-linear association (parabola)	450	**0.051** [0.037, 0.065]	**0.759** [0.732, 0.786]	**0.856** [0.834, 0.878]
non-linear association (parabola)	800	0.010 [0.003, 0.017]	0.969 [0.958, 0.980]	0.993 [0.987, 0.999]
non-linear association (wave)	125	0.253 [0.226, 0.280]	0.348 [0.318, 0.378]	0.193 [0.168, 0.218]
non-linear association (wave)	450	**0.152** [0.129, 0.175]	**0.863** [0.841, 0.885]	**0.841** [0.818, 0.864]
non-linear association (wave)	800	0.108 [0.088, 0.128]	0.992 [0.986, 0.998]	0.990 [0.983, 0.997]
weak positive association	125	0.278 [0.250, 0.306]	0.143 [0.121, 0.165]	0.072 [0.055, 0.089]
weak positive association	450	**0.257** [0.229, 0.285]	**0.182** [0.158, 0.206]	**0.183** [0.159, 0.207]
weak positive association	800	0.235 [0.208, 0.262]	0.316 [0.287, 0.345]	0.351 [0.321, 0.381]

Data simulated for a four-gene module.

^⋆^ estimate [95% confidence interval]

In view of these results, we choose to use the GLM-based Larntz & Perlman test in the DILGOM analysis.

### 4.2 DILGOM analysis

**Fig 5:**
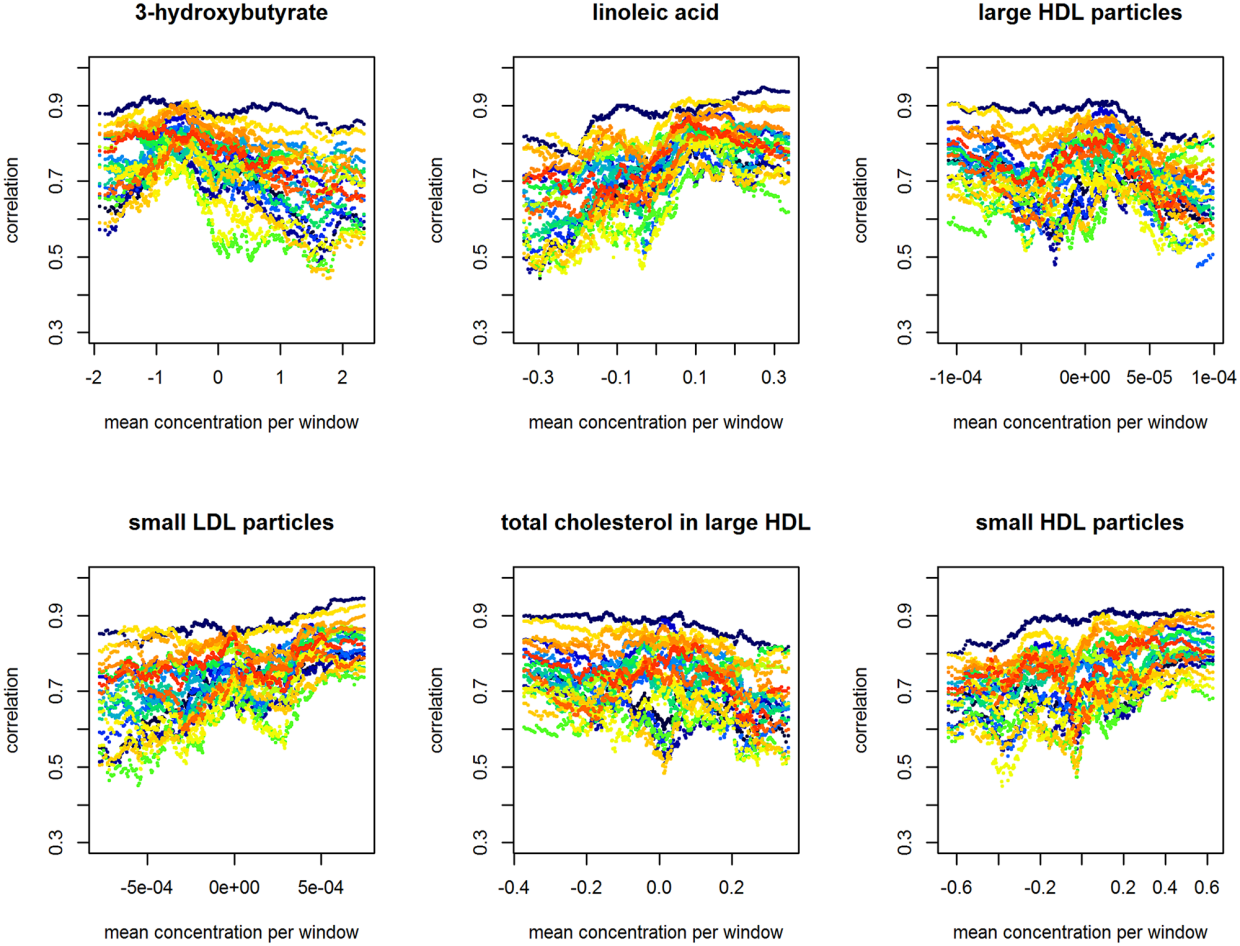
Co-expression dynamics by mean metabolic concentration based on sliding-window correlation estimates (*w* = 0.2). The *G* = 7 genes of the core LL module result in 21 gene-pair correlations. Each trajectory roughly captures the co-expression dynamics of one of the module’s gene pairs.

Fig 5 illustrates the changes in co-expression as a continuous function of the metabolic concentrations for the six metabolites: 3-hydroxybutyrate, linoleic acid, large HDL particles, small HDL particles, small LDL particles, and total cholesterol in large HDL; these are the results of the sliding-window procedure (Section 3.1). Evidently, the metabolite-co-expression relationship is not always monotonic, for instance, as see in the plots for 3-hydroxybutyrate, linoleic acid or large HDL particles.

**Fig 6:**
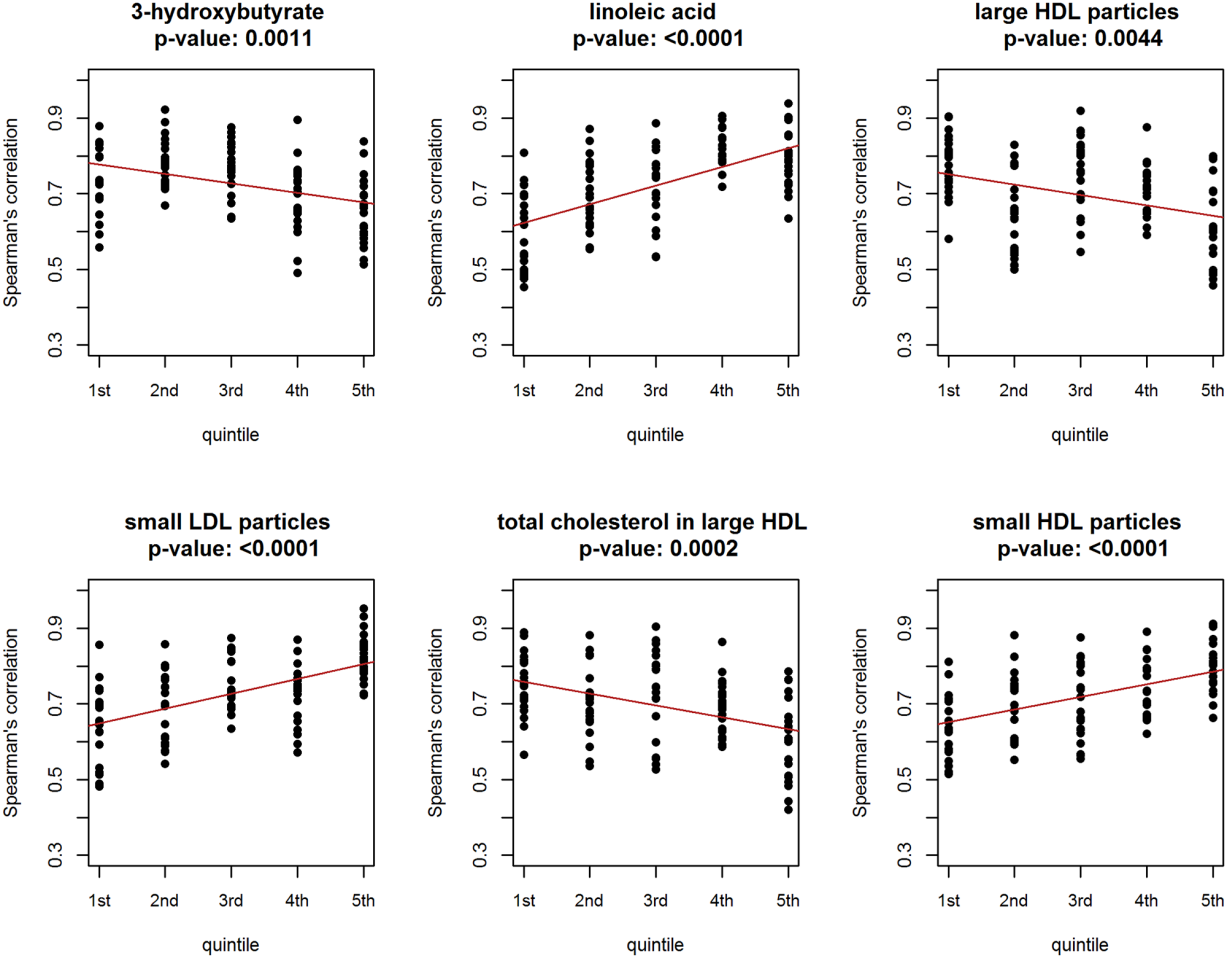
Results of the linear-regression-based investigation of conditional co-expression. Dots represent the estimated Spearman’s correlation coefficients for the five metabolic subsets (defined by quintiles of the metabolite); the fitted regression line is drawn in red. Benjamini and Yekutieli adjusted p-values are reported.

Fig 6 presents the results obtained by using the simple linear regression model for the six metabolites chosen for illustration. The adjusted *p*-values for all six metabolites suggest a statistically significant relationship between the correlation coefficients and the metabolite levels. Assuming a FDR of 5%, there are 80 metabolites (including the six presented in Fig 6) for which a metabolite-dependent co-expression could be concluded. However, given the results shown in Table 2, it is plausible that the linear-regression-based test is liberal in this case. Thus, in turn, we cannot be sure that the FDR is indeed controlled at the 5% level.

**Fig 7:**
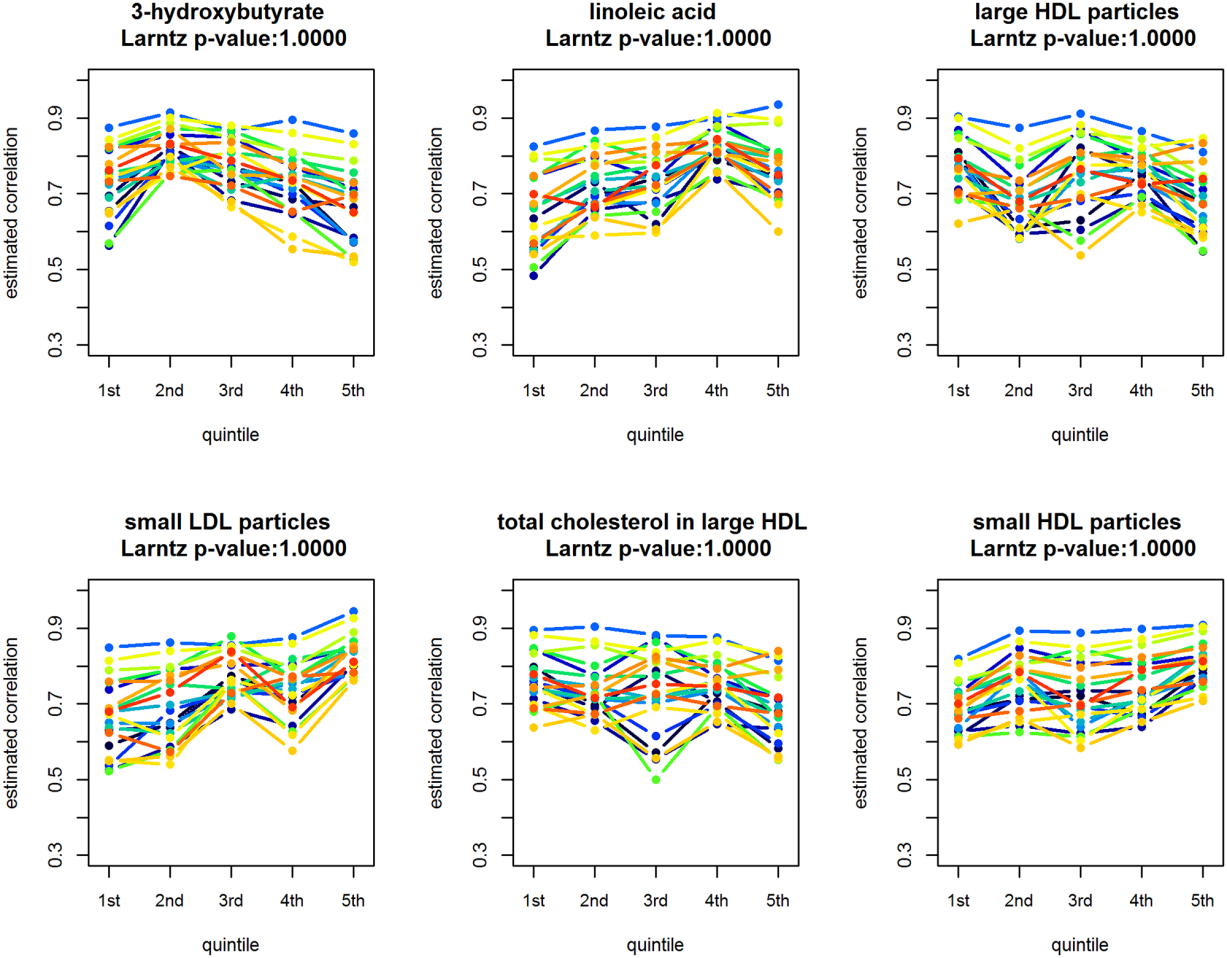
GLM based gene-pair correlation estimates for the five metabolic subsets. The estimates corresponding to a particular pair of genes are connected by a line. Benjamini and Yekutieli adjusted Larntz & Perlman test *p*-values are reported.

Fig 7 shows the metabolic-subset specific correlation between gene-pairs estimated using the GLM defined by Eqs (2) and (3). Based on the multiplicity-adjusted p-values of the Larntz & Perlman test, a statistically significant relationship between the co-expression and metabolite levels cannot be concluded for any of the metabolites. Given that the Larntz & Perlman test provides a proper control of the Type I error probability, we can expect that, in the analysis, the FDR is controlled at the 5% level.

**Fig 8:**
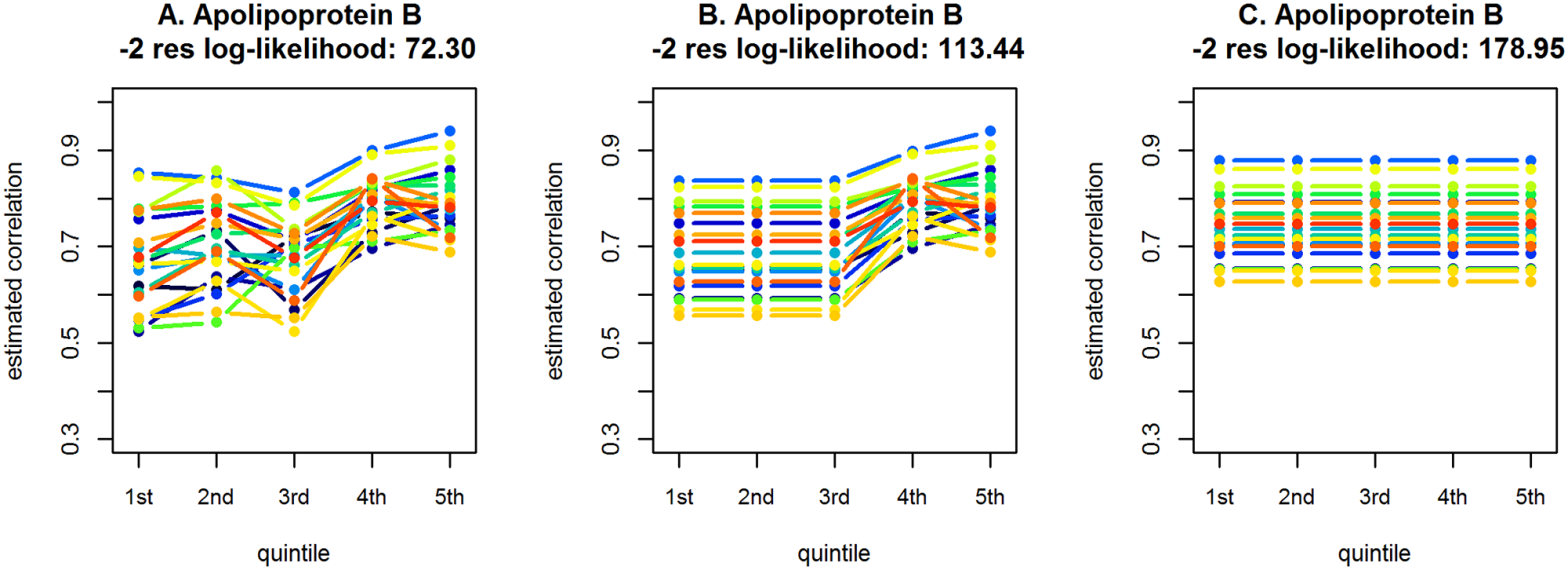
Estimated correlation coefficients, obtained using the general linear model with different variance-covariance structures, for the five metabolic subsets defined for apolipoprotein B. A. GLM with metabolic-subset specific correlation coefficients defined by Eqs (2) and (3); B. GLM with common correlation coefficients across the first three metabolic-subsets; C. GLM with no metabolic-subset dependent correlation coefficients, i.e., the null model defined by Eqs (2) and (4).

The GLM-framework is flexible in that it allows, for instance, the testing of a variety of hypotheses regarding the variance-covariance structure. To illustrate this aspect of the model, we use the concentration of apolipoprotein B as a potential mediator of the core LL module co-expression. The left-hand-side plot of Fig 8 presents the estimated correlation coefficients obtained using the GLM with the variance-covariance structure defined in Eq (3) with *S* = 5. We can see that the coefficients seem to only slightly deviate from a common value across the first three subsets (quintiles of the metabolite), while they seem to increase for the last two subsets. Using the Larntz and Perlman statistic, we can formally test whether a common correlation-coefficient could be assumed for the first three subsets. To this aim, we test each hypothesis of *H*_*g*_1_*g*_2__ : *ρ*1, _*g*_1_*g*_2__ = *ρ*2, _*g*_1_*g*_2__ = *ρ*3, _*g*_1_*g*_2__, for all *g*_1_ ≠ *g*_2_ (*g*_1_, *g*_2_ = 1, …, *G*). The result of the Larntz & Perlman test is not statistically significant (*p* = 0.9950), suggesting that the simpler variance-covariance structure might be adopted. The plot in the middle column of Fig 8 presents the estimated correlation coefficients based on the simplified model. In turn, one could compare the correlation matrices of the simpler model to test for a difference between metabolic subsets, i.e., to determine whether the GLM with the variance-covariance structure defined in Eq (4) can be adopted. The right-hand-side plot of Fig 8 presents the estimates of the correlation coefficients obtained for the GLM defined by Eqs (2) and (4). The result of the corresponding Larntz & Perlman test is statistically significant (*p* = 0.0079), suggesting that the observed increase of the correlation coefficients across the last two subsets cannot be attributed to a chance variation. The aforementioned results are data-driven and do not take into account the multiple-testing adjustment, but they do illustrate the potential of the GLM in testing various hypotheses that might be of interest.

## 5 Discussion

The use of the GLM offers a formal, flexible framework to investigate the co-expression-mediation of a gene module. The model facilitates the adjustment of gene expression values for any potential confounding factors. Questions regarding the conditional co-expression can be formulated as hypotheses about the variance-covariance structure of gene expression measurements and formally tested by using the Larntz & Perlman test or the LR test (provided that, for the latter, an adequate sample size is available). The model can be fitted using existing software like SAS (PROC MIXED or PROC GLIMMIX) [9, 10].

As compared to the approach proposed by [7], the GLM-based analysis requires the assumption of normality of the gene expression measurements. One can see it as a drawback. However, models based on such an assumption (assumed, often, on the logarithmic scale) have already been considered in the literature [22–24]. Assessing all aspects of multivariate normality is difficult. However, investigating univariate normality, though it will not guarantee multivariate normality, can detect cases of multivariate non-normality. Quantile-quantile plots of the GLM residuals were used to assess the univariate normality (see S1–S6 Figs). In this way, the plausibility of the assumption can be checked. In return, the GLM-based approach removes the limitations (Eqs 1–3) of the linear-regression-based analysis mentioned in Section 1.

The advantages of using a formal modeling framework were illustrated in the simulation study and in the analysis of the metabolite-mediated conditional co-expression of the core LL gene module. Worth noting is the fact that we did not identify any statistically significant metabolite-co-expression associations. The linear-regression approach results in 80 such associations. This large discrepancy is not surprising in light of the simulation study. For a seven-gene module and a sample size of *n* = 450 observations, the simulation study indicated that the linear-regression approach fails to control the Type I error probability (SLR: 0.314 [0.285, 0.343] vs. GLM-based Larntz & Perlman test: 0.036 [0.024, 0.048]). In a linear regression model, inconsistent standard error estimates may arise as a consequence of ignoring any estimation error inherent in the dependent variable [25]. The regression approach ignores the estimation error in the observed correlation coefficients. In addition, the coefficients estimated for the same metabolic subset are treated as independent, though they are not. Consequently, the precision of the estimation of the linear regression coefficients may be overestimated, resulting in too small raw *p*-values and an excess of “false positive” findings even after a multiple-testing correction.

A potential issue in the use of the GLM approach is the number of parameters. Besides the coefficients used in the mean-structure Eq (2), the most general variance-covariance structure Eq (3) involves *SG* variances and *SG*(*G* − 1)/2 correlation coefficients, i.e., *SG*(*G* + 1)/2 parameters. Depending on the size of the gene module and the number of metabolic subsets, the number can be very large. For instance, for the core LL gene module with *G* = 7 genes and *S* = 5 subsets, the number of variance-covariance parameters is equal to 140. Thus, estimation of the model requires a considerable sample size. Note, however, that the same remark applies to the linear-regression approach, as it also requires estimation of the *SG*(*G* − 1)/2 correlation coefficients (105 in the case of the core LL gene module).

Another drawback shared by the linear-regression and GLM approaches is that they require the splitting of the metabolite measurements into subsets. Naturally, this implies that the results may depend on the definition of the subsets. A possible solution to this problem would be to model the correlation coefficients as a function of metabolite values. One could imagine using a suitable class of functions, capturing the trends seen in Fig 5, to model the correlation coefficients in the variance-covariance matrix Eq (3). Such a solution would obviate the need for defining metabolic subsets. This is a topic of current research.

## Supporting Information

S1 File Design matrix *X*_*si*_ of Eq 2. (PDF)

S1 SAS Code SAS procedure GLIMMIX GLM code for a gene-module comprised of three genes. (PDF)

S1 Table Type I error rates for the GLM-based test statistics by module size and sample size. (PDF)

S2 Table Power of the GLM-based test statistics for different co-expression dynamics and sample sizes. (PDF)

S1 Fig Univariate quantile-quantile plots of the GLM residuals for 3-hydroxybutyrate. (TIFF)

S2 Fig Univariate quantile-quantile plots of the GLM residuals for linoleic acid. (TIFF)

S3 Fig Univariate quantile-quantile plots of the GLM residuals for large HDL particles. (TIFF)

S4 Fig Univariate quantile-quantile plots of the GLM residuals for small LDL particles. (TIFF)

S5 Fig Univariate quantile-quantile plots of the GLM residuals for total cholesterol in large HDL. (TIFF)

S6 Fig Univariate quantile-quantile plots of the GLM residuals for small HDL particles. (TIFF)
