# Supplementary material for: The Detection of Metabolite-Mediated Gene Module Co-Expression Using Multivariate Linear Models
Source: PLoS One. 2016 Feb 26;11(2):e0150257. doi: 10.1371/journal.pone.0150257 (PMC4769021; doi:10.1371/journal.pone.0150257)
Supplement: S1 File — (PDF) [file pone.0150257.s001.pdf]

Supplementary Material

An example of the design matrix, X_{si}, for the i-th individual in the s-th subset (see Equation 2 of Section 3.3) is provided below. This representation is based on a model including the covariates: gene, metabolic concentration, age, gender and the two-way interaction between gene and metabolic concentrations.

$$
\begin{array}{cccccccccccc}
\mu & g_1 & g_2 & \cdots & g_{G-1} & conc. & age & gender & g_1 * conc. & g_2 * conc. & \cdots & g_{G-1} * conc.
\end{array}
$$

$$
X_{si} = \begin{bmatrix}
1 & 1 & 0 & \cdots & 0 & 0.3 & 30 & 1 & 0.3 & 0 & \cdots & 0 \\
1 & 0 & 1 & \cdots & 0 & 0.3 & 30 & 1 & 0 & 0.3 & \cdots & 0 \\
\vdots & \vdots & \vdots & \vdots & \vdots & \vdots & \vdots & \vdots & \vdots & \vdots & \vdots & \vdots \\
1 & 0 & 0 & \cdots & 1 & 0.3 & 30 & 1 & 0 & 0 & \cdots & 0.3 \\
1 & 0 & 0 & \cdots & 0 & 0.3 & 30 & 1 & 0 & 0 & \cdots & 0
\end{bmatrix}
$$
