# Supplementary material for: The Detection of Metabolite-Mediated Gene Module Co-Expression Using Multivariate Linear Models
Source: PLoS One. 2016 Feb 26;11(2):e0150257. doi: 10.1371/journal.pone.0150257 (PMC4769021; doi:10.1371/journal.pone.0150257)
Supplement: S1 SAS Code — (PDF) [file pone.0150257.s002.pdf]

SAS PROC GLIMMIX GLM CODE FOR A GENE-MODULE COMPRISED OF THREE GENES (Padayachee et al. 2015)

```
proc GLIMMIX data=DATA;
* DATA is in long format, sorted by m_INDEX, SUBJECT and GENE;
* GE is the vector of gene expression values;
* adjMETABOLITE is the adjusted metabolic concentrations;

  ods select none;
  by m_INDEX;
  nloptions TECHNIQUE = NRRIDG;
  class SUBJECT GENE GENDER m_QUINTILE;
  model GE = AGE|GENE|adjMETABOLITE|GENDER;
  random _residual_ / subject = SUBJECT type=unr group = m_QUINTILE;

 * OPTIONAL: Input initial values using the PARMS statement to solve convergence problems;

  covtest "test for homogeneous correlations" general
0 0 0 1 0 0 0 0 0 -1 0 0 0 0 0 0 0 0 0 0 0 0 0 0 0 0 0 0 0 0 ,
0 0 0 0 1 0 0 0 0 0 -1 0 0 0 0 0 0 0 0 0 0 0 0 0 0 0 0 0 0 0 ,
0 0 0 0 0 1 0 0 0 0 0 -1 0 0 0 0 0 0 0 0 0 0 0 0 0 0 0 0 0 0 ,
0 0 0 1 0 0 0 0 0 0 0 0 0 0 0 -1 0 0 0 0 0 0 0 0 0 0 0 0 0 0 ,
0 0 0 0 1 0 0 0 0 0 0 0 0 0 0 0 -1 0 0 0 0 0 0 0 0 0 0 0 0 0 ,
0 0 0 0 0 1 0 0 0 0 0 0 0 0 0 0 0 -1 0 0 0 0 0 0 0 0 0 0 0 0 ,
0 0 0 1 0 0 0 0 0 0 0 0 0 0 0 0 0 0 0 0 0 -1 0 0 0 0 0 0 0 0 ,
0 0 0 0 1 0 0 0 0 0 0 0 0 0 0 0 0 0 0 0 0 0 -1 0 0 0 0 0 0 0 ,
0 0 0 0 0 1 0 0 0 0 0 0 0 0 0 0 0 0 0 0 0 0 0 -1 0 0 0 0 0 0 ,
0 0 0 1 0 0 0 0 0 0 0 0 0 0 0 0 0 0 0 0 0 0 0 0 0 0 0 -1 0 0 ,
0 0 0 0 1 0 0 0 0 0 0 0 0 0 0 0 0 0 0 0 0 0 0 0 0 0 0 0 -1 0 ,
0 0 0 0 0 1 0 0 0 0 0 0 0 0 0 0 0 0 0 0 0 0 0 0 0 0 0 0 0 -1 ;

   ods output FitStatistics = Fit
              CovParms = Estimates
                     CovTests = TestResults;
run;
```
