# Supplementary material for: The Detection of Metabolite-Mediated Gene Module Co-Expression Using Multivariate Linear Models
Source: PLoS One. 2016 Feb 26;11(2):e0150257. doi: 10.1371/journal.pone.0150257 (PMC4769021; doi:10.1371/journal.pone.0150257)
Supplement: S1 Table — (PDF) [file pone.0150257.s003.pdf]

Supplementary Table 1

Table 1: **Type I error probabilities for the GLM-based test statistics by module size and sample size**

**module size**	**sample size (n)**	**GLM-based LR test**⋆	**GLM-based Larntz & Perlman**⋆	**GLM-based Jennrich**⋆	**GLM-based Cole**⋆
module size	sample size	LRT type I error	Larntz type I error	Jennrich type I error	Cole type I error
4	125	0.109 [0.089, 0.129]	0.045 [0.032, 0.058]	0.091 [0.073, 0.109]	0.198 [0.173, 0.223]
4	450	0.062 [0.047, 0.077]	0.043 [0.030, 0.056]	0.065 [0.049, 0.081]	0.082 [0.064, 0.100]
4	800	0.053 [0.039, 0.067]	0.035 [0.023, 0.047]	0.050 [0.036, 0.064]	0.063 [0.047, 0.079]
5	125	0.141 [0.119, 0.163]	0.048 [0.034, 0.062]	0.091 [0.073, 0.109]	0.288 [0.259, 0.317]
5	450	0.067 [0.051, 0.083]	0.037 [0.025, 0.049]	0.055 [0.040, 0.070]	0.088 [0.070, 0.106]
5	800	0.066 [0.050, 0.082]	0.048 [0.034, 0.062]	0.054 [0.039, 0.069]	0.073 [0.056, 0.090]
7	125	0.314 [0.285, 0.343]	0.035 [0.023, 0.047]	0.104 [0.085, 0.123]	0.572 [0.541, 0.603]
7	450	0.083* [0.065, 0.100]	0.036* [0.024, 0.048]	0.068* [0.051, 0.084]	0.089* [0.071, 0.107]
7	800	0.070** [0.054, 0.087]	0.029** [0.018, 0.040]	0.058** [0.043, 0.073]	0.080** [0.062, 0.097]

⋆ estimate [95% confidence interval]

∗ convergence rate of GLM: 0.991

∗∗ convergence rate of GLM: 0.993
