# Supplementary material for: The Detection of Metabolite-Mediated Gene Module Co-Expression Using Multivariate Linear Models
Source: PLoS One. 2016 Feb 26;11(2):e0150257. doi: 10.1371/journal.pone.0150257 (PMC4769021; doi:10.1371/journal.pone.0150257)
Supplement: S2 Table — (PDF) [file pone.0150257.s004.pdf]

Supplementary Table 2

Table 2: **Power of the GLM-based test statistics for different co-expression dynamics and sample sizes**

**co-expression dynamics**	**sample size (n)**	**GLM-based LR test***	**GLM-based Larntz & Perlman***	**GLM-based Jennrich***	**GLM-based Cole***
approx. linear positive association	125	0.314 [0.285, 0.343]	0.188 [0.163, 0.213]	0.239 [0.212, 0.266]	0.374 [0.344, 0.404]
approx. linear positive association	450	0.826 [0.802, 0.850]	0.797 [0.772, 0.822]	0.711 [0.682, 0.740]	0.690 [0.661, 0.719]
approx. linear positive association	800	0.990 [0.983, 0.997]	0.989 [0.982, 0.996]	0.962 [0.950, 0.974]	0.953 [0.939, 0.967]
approx. linear negative association	125	0.300 [0.271, 0.329]	0.184 [0.159, 0.209]	0.228 [0.201, 0.255]	0.358 [0.328, 0.388]
approx. linear negative association	450	0.838 [0.815, 0.861]	0.819 [0.795, 0.843]	0.716 [0.688, 0.744]	0.693 [0.664, 0.722]
approx. linear negative association	800	0.988 [0.981, 0.995]	0.987 [0.979, 0.995]	0.960 [0.947, 0.973]	0.949 [0.935, 0.963]
non-linear association	125	0.293 [0.264, 0.322]	0.243 [0.216, 0.270]	0.240 [0.213, 0.267]	0.457 [0.426, 0.488]
non-linear association	450	0.759 [0.732, 0.786]	0.856 [0.834, 0.878]	0.744 [0.716, 0.772]	0.748 [0.721, 0.775]
non-linear association	800	0.969 [0.958, 0.980]	0.993 [0.987, 0.999]	0.963 [0.951, 0.975]	0.960 [0.947, 0.973]
non-linear association	125	0.348 [0.318, 0.378]	0.193 [0.168, 0.218]	0.228 [0.201, 0.255]	0.371 [0.341, 0.401]
non-linear association	450	0.863 [0.841, 0.885]	0.841 [0.818, 0.864]	0.693 [0.664, 0.722]	0.648 [0.618, 0.678]
non-linear association	800	0.992 [0.986, 0.998]	0.990 [0.983, 0.997]	0.956 [0.943, 0.969]	0.943 [0.928, 0.958]
weak positive association	125	0.143 [0.121, 0.165]	0.072 [0.055, 0.089]	0.131 [0.110, 0.152]	0.267 [0.239, 0.295]
weak positive association	450	0.182 [0.158, 0.206]	0.183 [0.159, 0.207]	0.192 [0.167, 0.217]	0.205 [0.179, 0.231]
weak positive association	800	0.316 [0.287, 0.345]	0.351 [0.321, 0.381]	0.342 [0.312, 0.372]	0.340 [0.310, 0.370]

Data simulated for a four-gene module.

⋆ estimate [95% confidence interval]
