# Supplementary figures and images for: The Detection of Metabolite-Mediated Gene Module Co-Expression Using Multivariate Linear Models
Source: PLoS One. 2016 Feb 26;11(2):e0150257. doi: 10.1371/journal.pone.0150257 (PMC4769021; doi:10.1371/journal.pone.0150257)

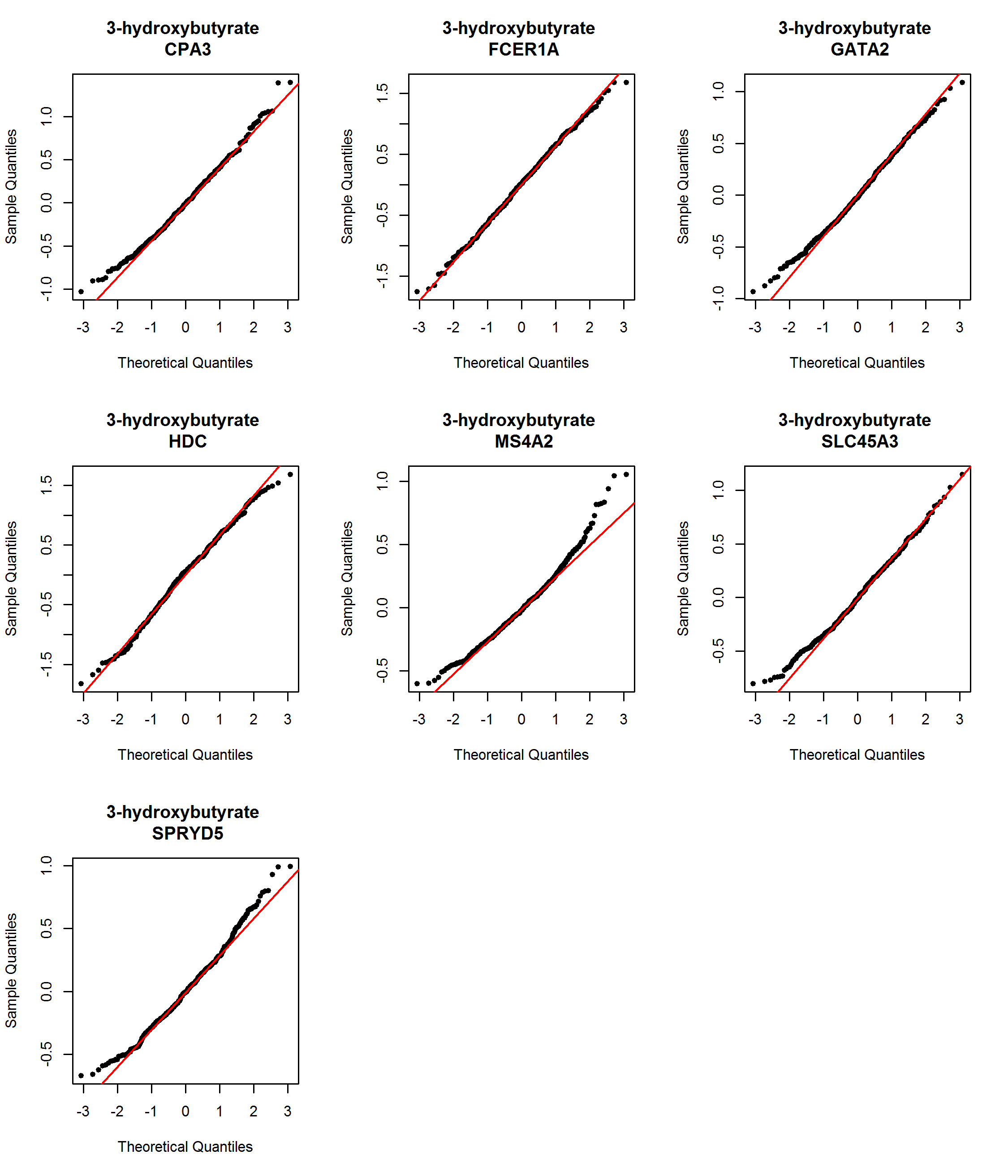

Supplement: S1 Fig — (TIFF) [file pone.0150257.s005.tiff]

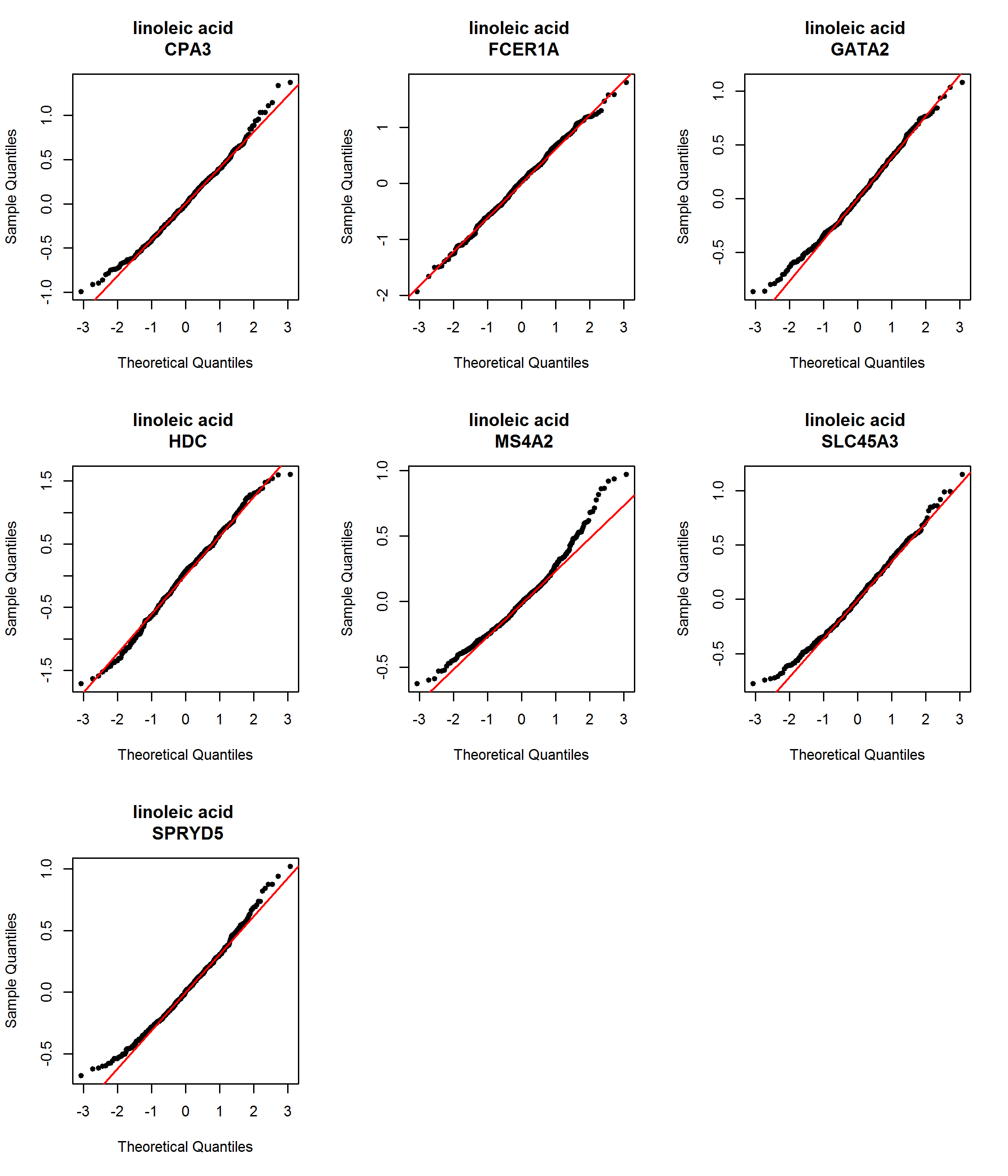

Supplement: S2 Fig — (TIFF) [file pone.0150257.s006.tiff]

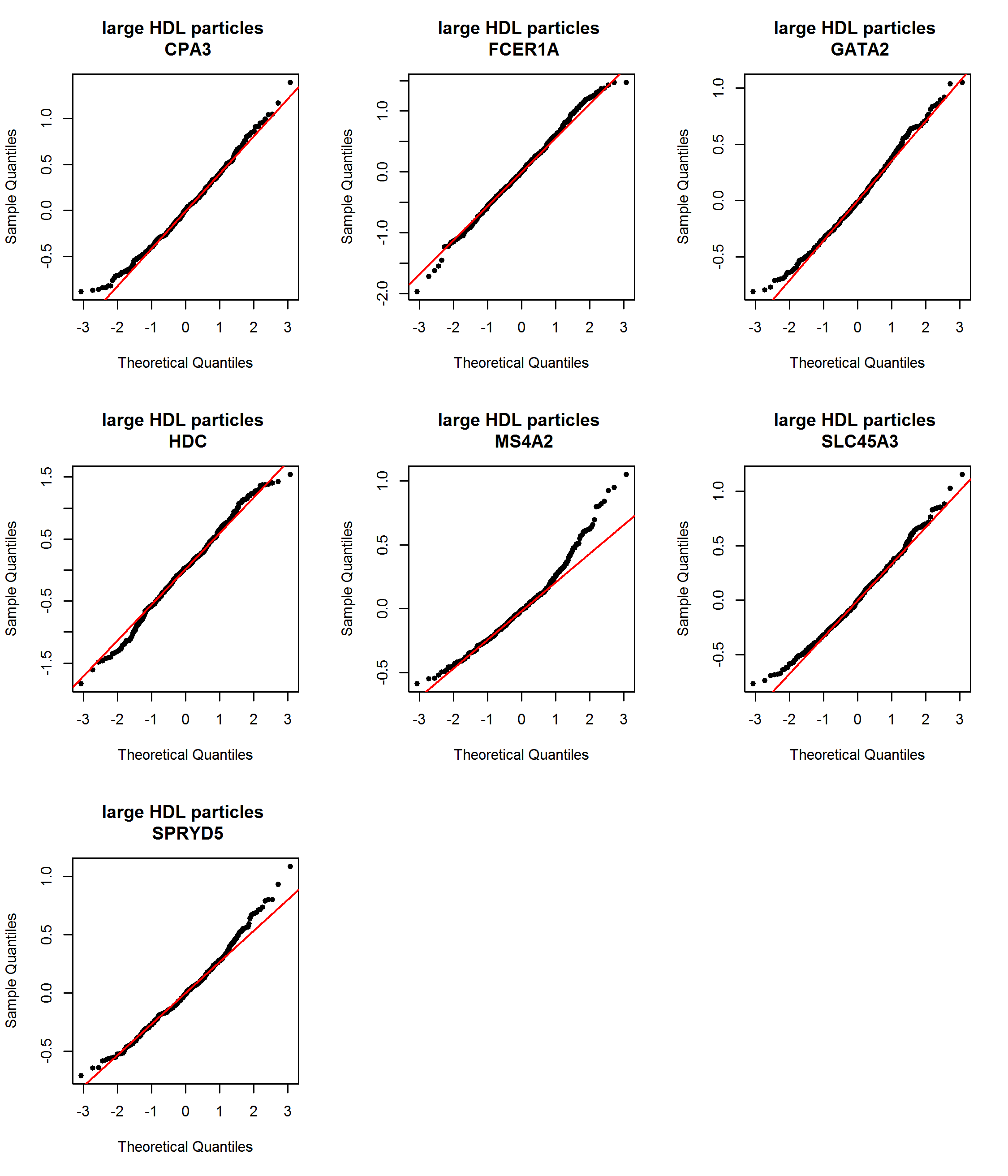

Supplement: S3 Fig — (TIFF) [file pone.0150257.s007.tiff]

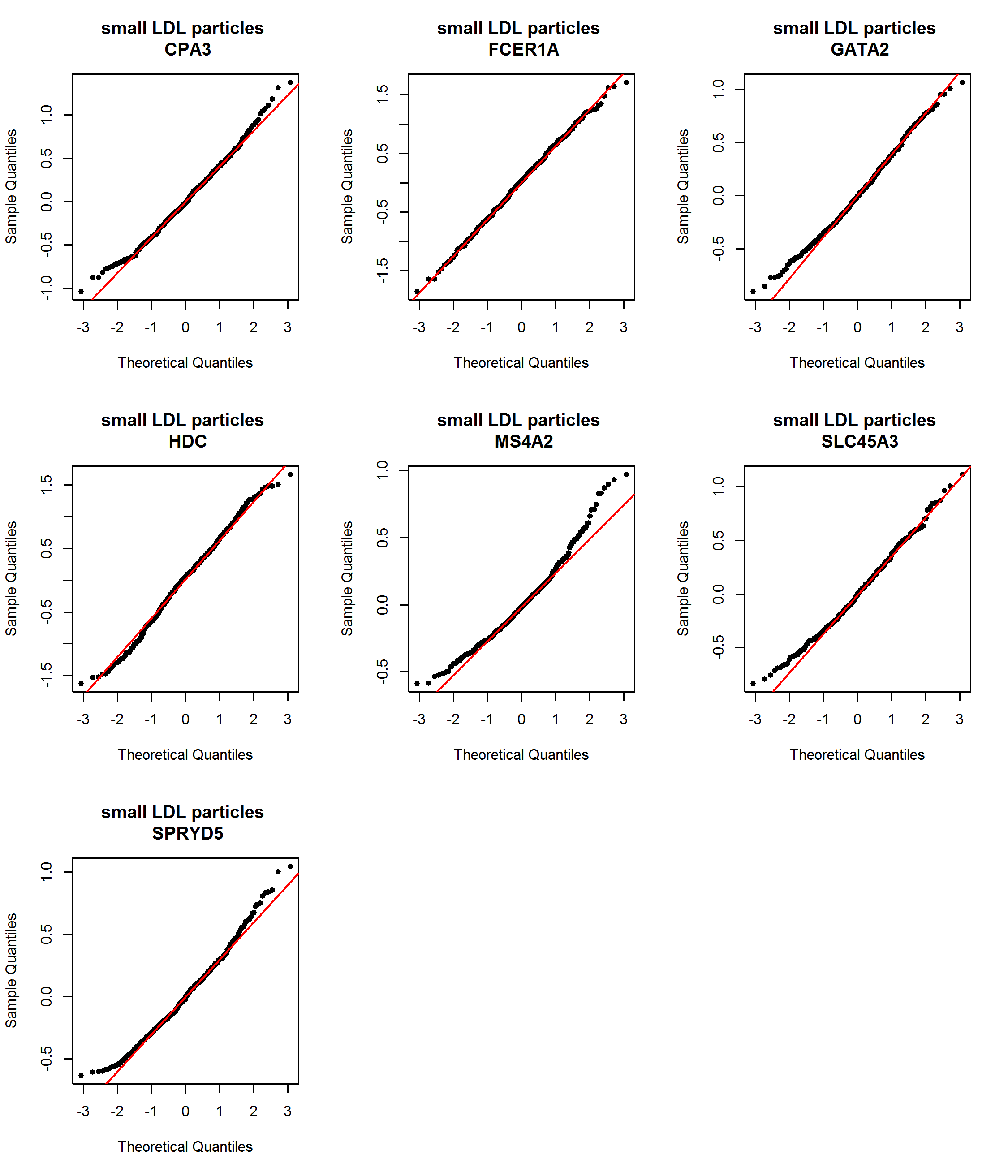

Supplement: S4 Fig — (TIFF) [file pone.0150257.s008.tiff]

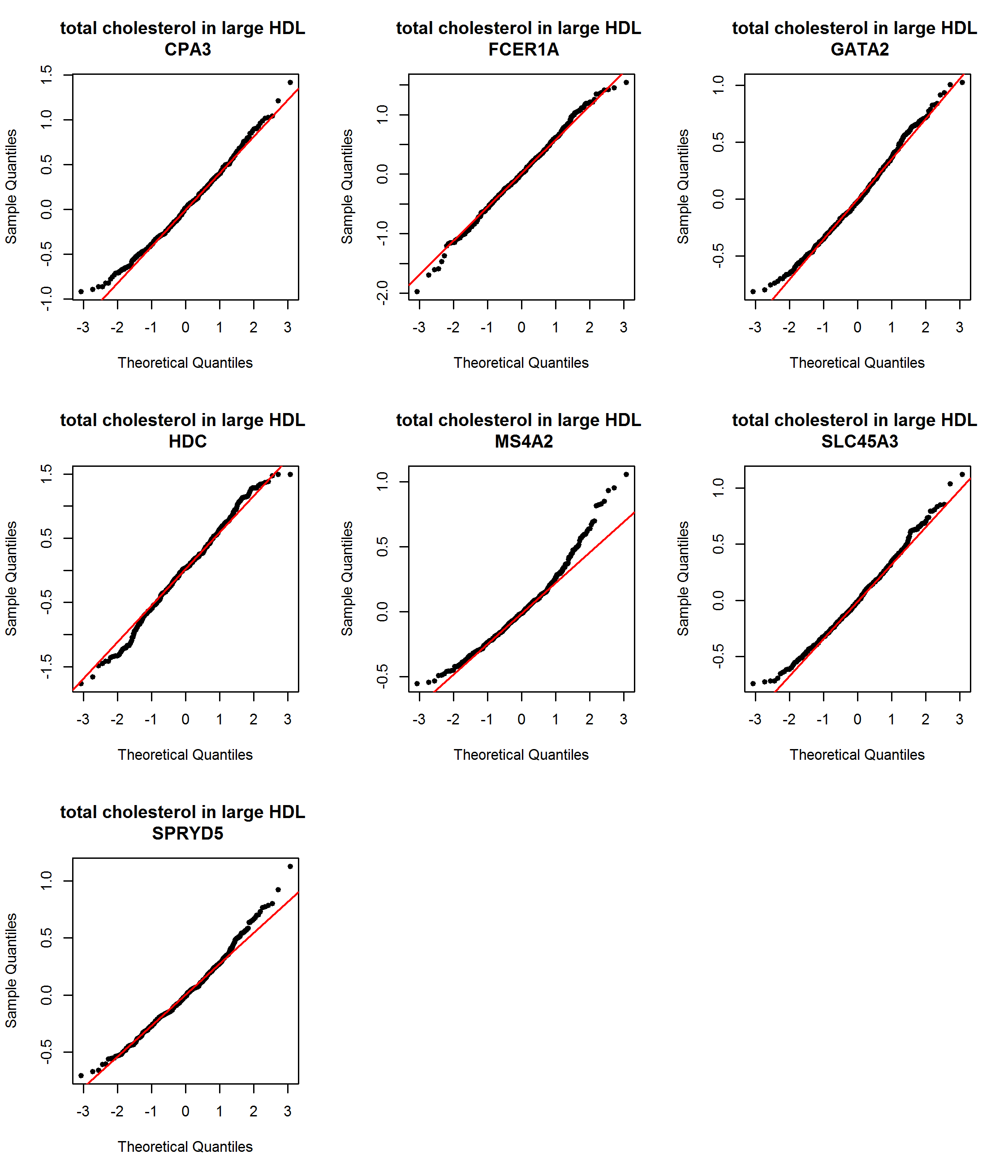

Supplement: S5 Fig — (TIFF) [file pone.0150257.s009.tiff]

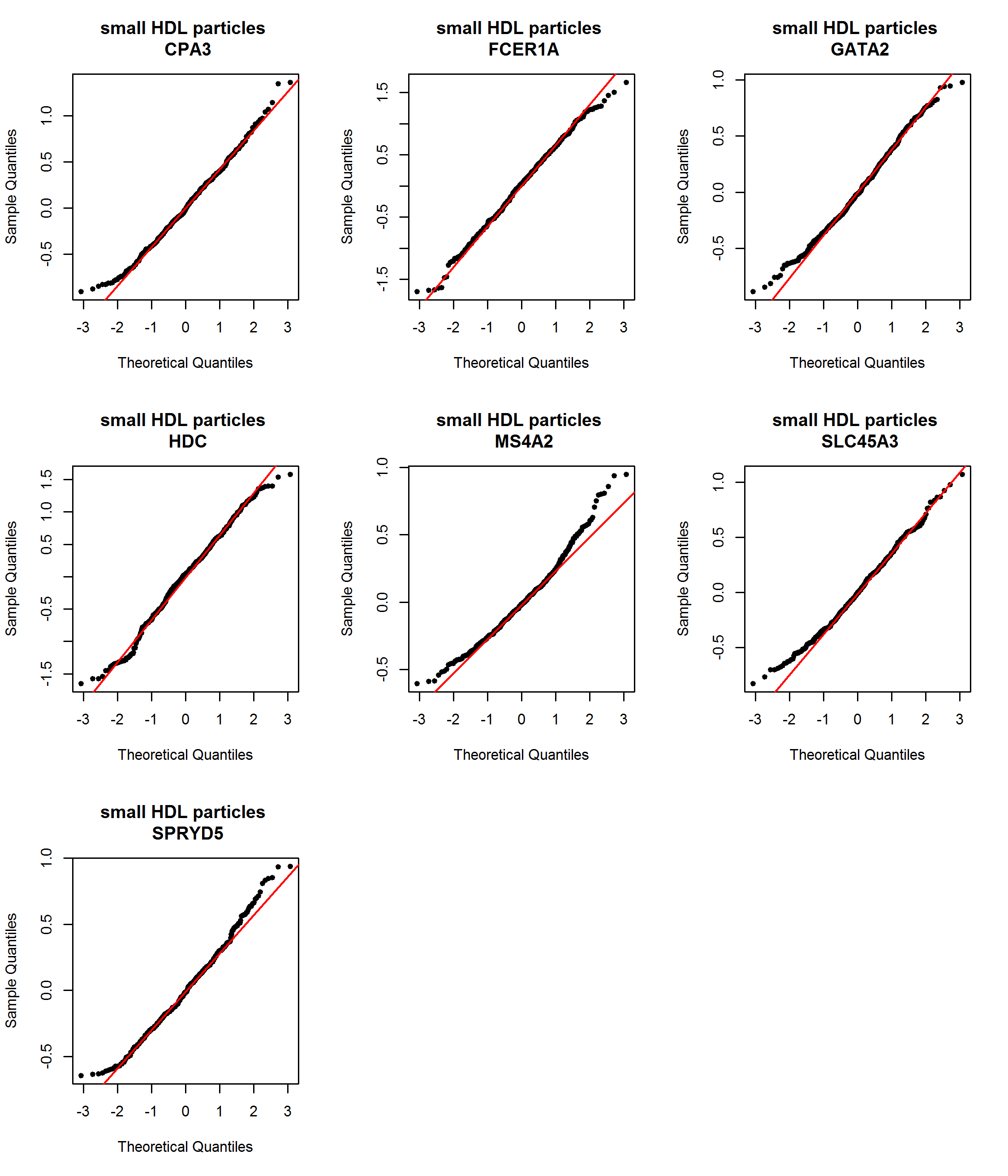

Supplement: S6 Fig — (TIFF) [file pone.0150257.s010.tiff]
